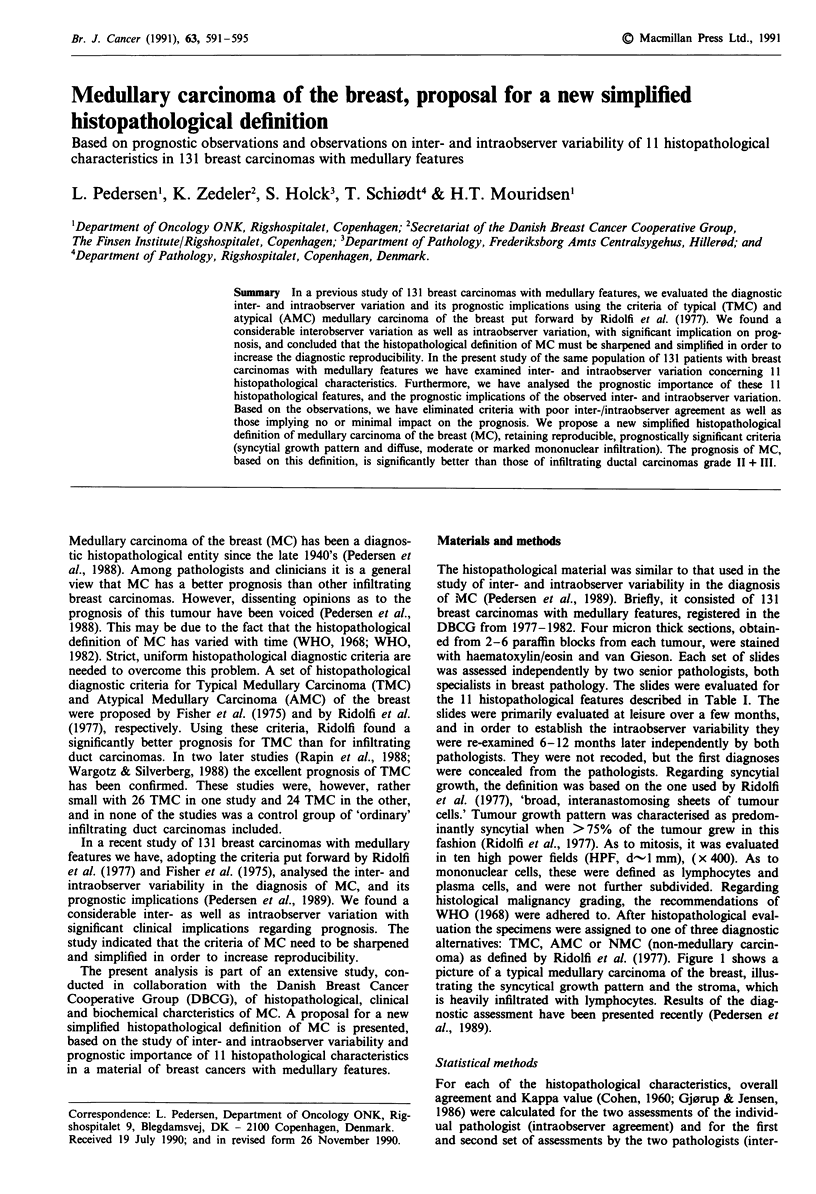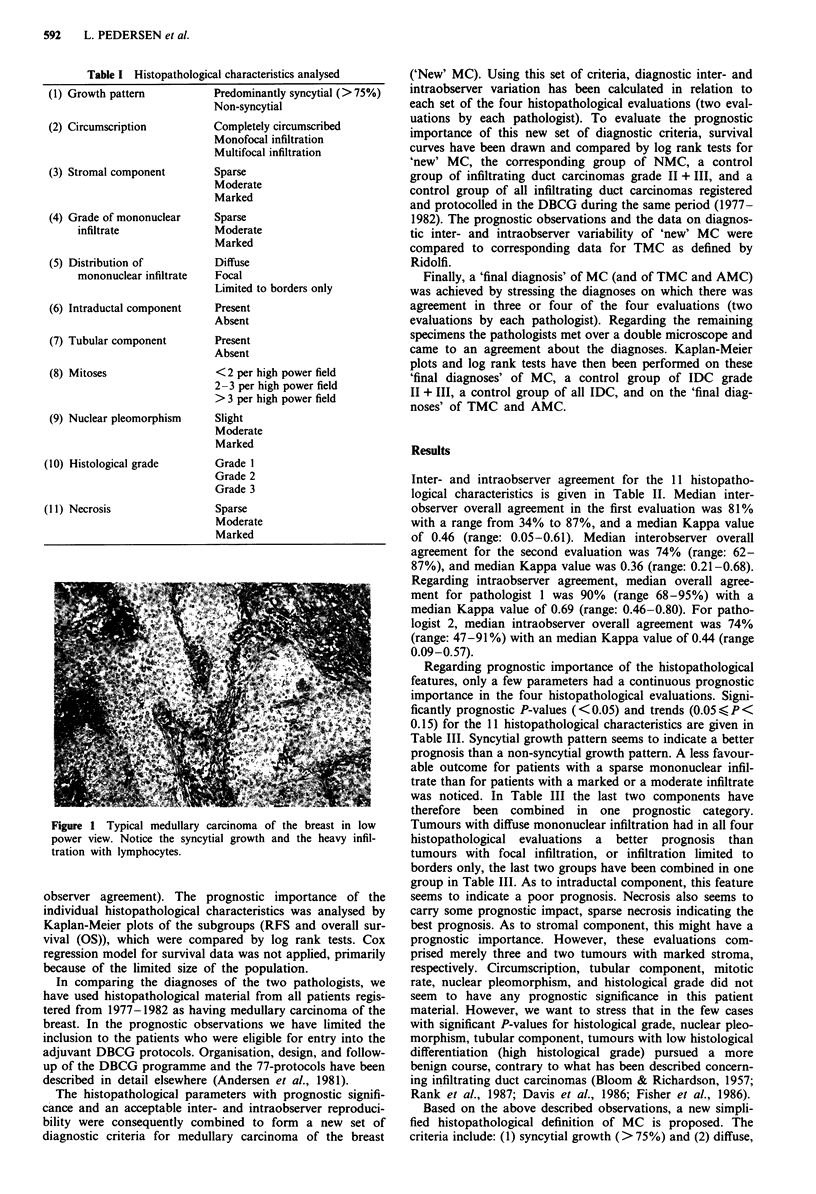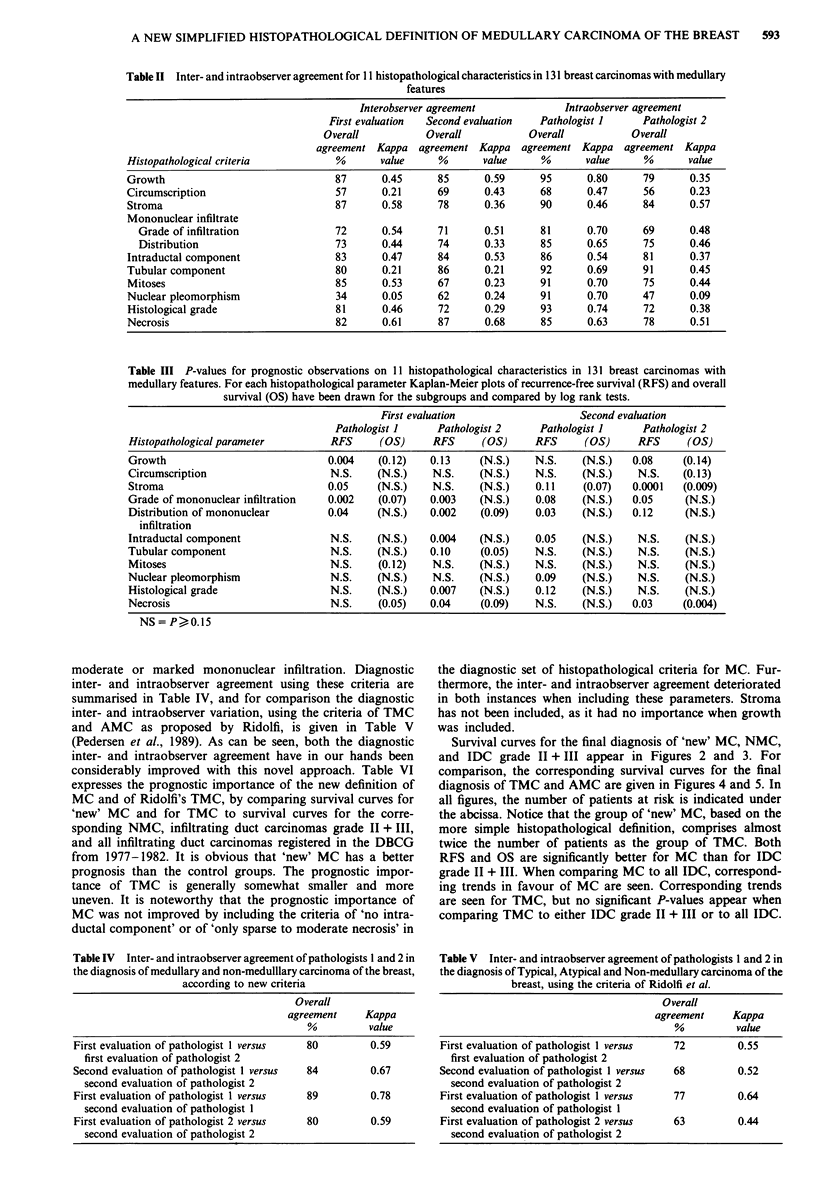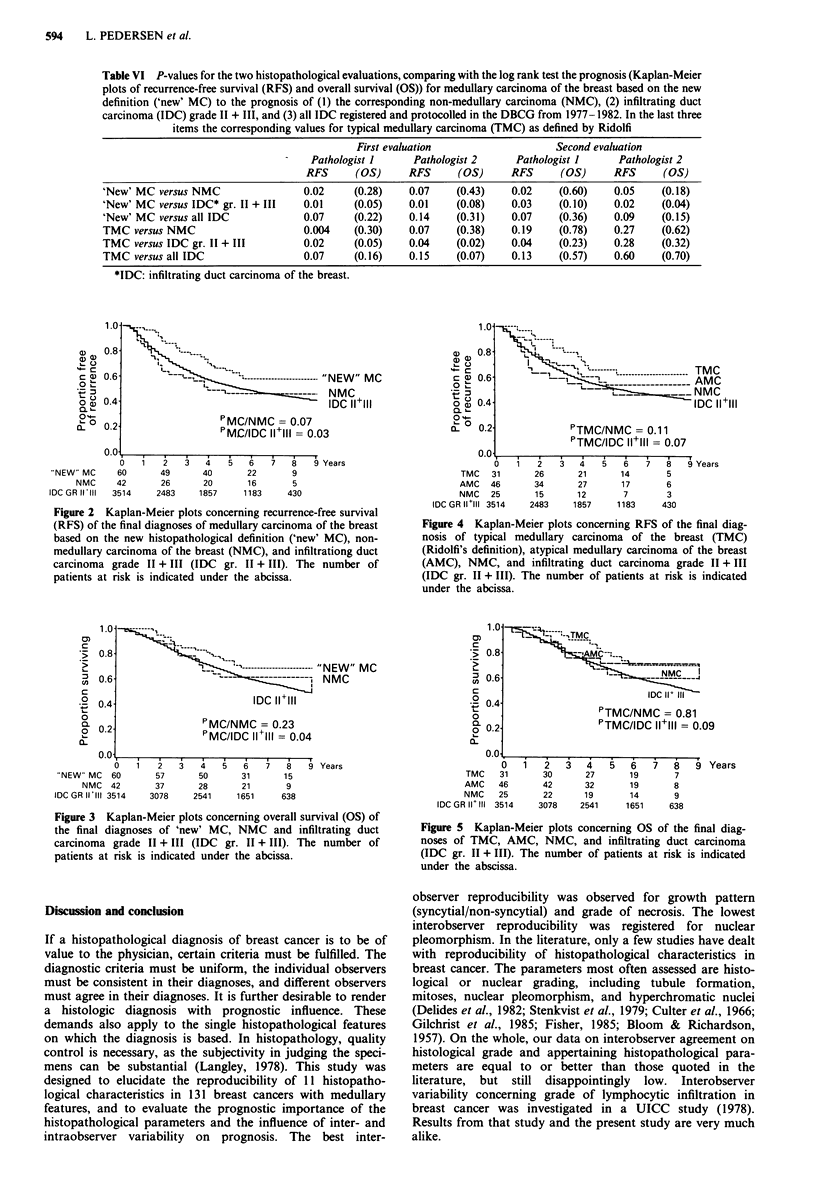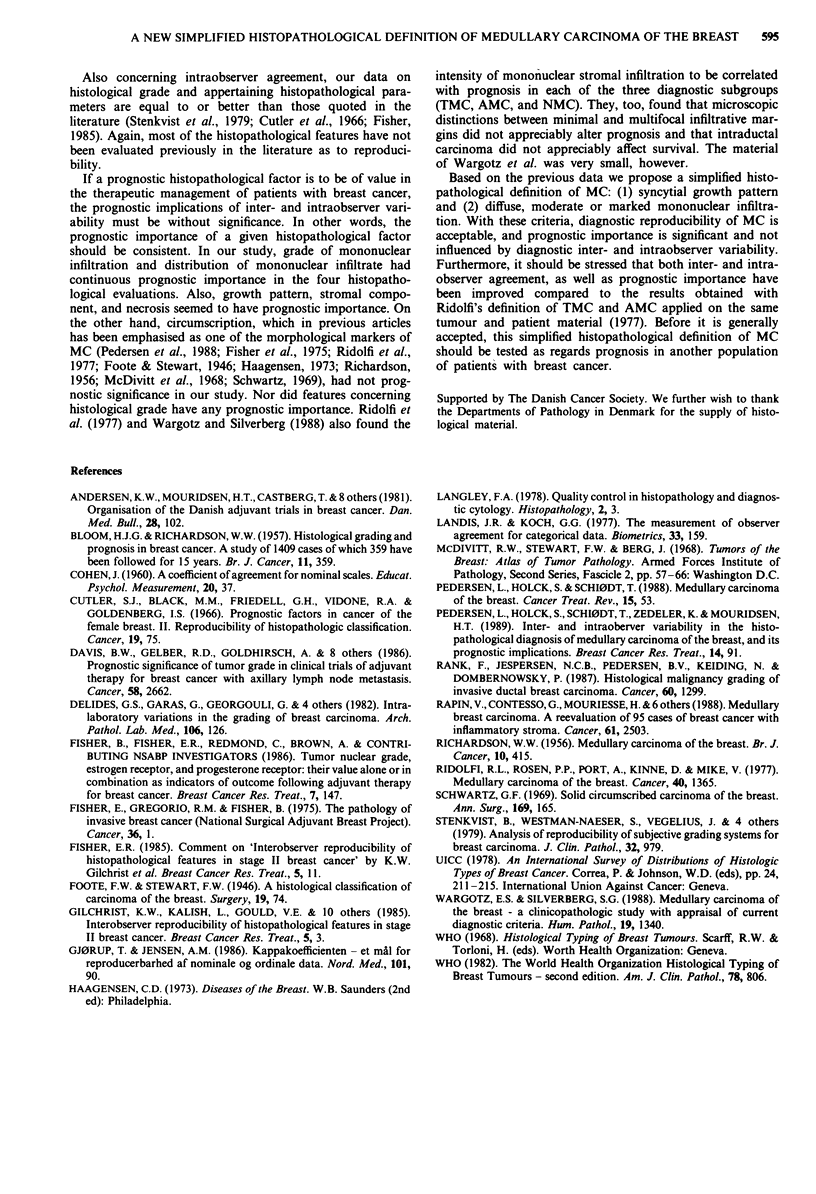# Medullary carcinoma of the breast, proposal for a new simplified histopathological definition. Based on prognostic observations and observations on inter- and intraobserver variability of 11 histopathological characteristics in 131 breast carcinomas with medullary features.

**DOI:** 10.1038/bjc.1991.137

**Published:** 1991-04

**Authors:** L. Pedersen, K. Zedeler, S. Holck, T. Schiødt, H. T. Mouridsen

**Affiliations:** Department of Oncology ONK, Rigshospitalet, Copenhagen, Denmark.

## Abstract

**Images:**


Br. J. Cancer (1991), 63, 591–595 © Macmillan Press Ltd., 1991

### Medullary carcinoma of the breast, proposal for a new simplified histopathological definition

Based on prognostic observations and observations on inter- and intraobserver variability of 11 histopathological characteristics in 131 breast carcinomas with medullary features

L. Pedersen[1], K. Zedeler[2], S. Holck[3], T. Schiødt[4] & H.T. Mouridsen[1]

[1]*Department of Oncology ONK, Rigshospitalet, Copenhagen;* [2]*Secretariat of the Danish Breast Cancer Cooperative Group, The Finsen Institute/Rigshospitalet, Copenhagen;* [3]*Department of Pathology, Frederiksborg Amts Centralsygehus, Hillerød; and* [4]*Department of Pathology, Rigshospitalet, Copenhagen, Denmark.*

**Summary** In a previous study of 131 breast carcinomas with medullary features, we evaluated the diagnostic inter- and intraobserver variation and its prognostic implications using the criteria of typical (TMC) and atypical (AMC) medullary carcinoma of the breast put forward by Ridolfi *et al.* (1977). We found a considerable interobserver variation as well as intraobserver variation, with significant implication on prognosis, and concluded that the histopathological definition of MC must be sharpened and simplified in order to increase the diagnostic reproducibility. In the present study of the same population of 131 patients with breast carcinomas with medullary features we have examined inter- and intraobserver variation concerning 11 histopathological characteristics. Furthermore, we have analysed the prognostic importance of these 11 histopathological features, and the prognostic implications of the observed inter- and intraobserver variation. Based on the observations, we have eliminated criteria with poor inter-/intraobserver agreement as well as those implying no or minimal impact on the prognosis. We propose a new simplified histopathological definition of medullary carcinoma of the breast (MC), retaining reproducible, prognostically significant criteria (syncytial growth pattern and diffuse, moderate or marked mononuclear infiltration). The prognosis of MC, based on this definition, is significantly better than those of infiltrating ductal carcinomas grade II + III.

Medullary carcinoma of the breast (MC) has been a diagnostic histopathological entity since the late 1940's (Pedersen *et al.*, 1988). Among pathologists and clinicians it is a general view that MC has a better prognosis than other infiltrating breast carcinomas. However, dissenting opinions as to the prognosis of this tumour have been voiced (Pedersen *et al.*, 1988). This may be due to the fact that the histopathological definition of MC has varied with time (WHO, 1968; WHO, 1982). Strict, uniform histopathological diagnostic criteria are needed to overcome this problem. A set of histopathological diagnostic criteria for Typical Medullary Carcinoma (TMC) and Atypical Medullary Carcinoma (AMC) of the breast were proposed by Fisher *et al.* (1975) and by Ridolfi *et al.* (1977), respectively. Using these criteria, Ridolfi found a significantly better prognosis for TMC than for infiltrating duct carcinomas. In two later studies (Rapin *et al.*, 1988; Wargotz & Silverberg, 1988) the excellent prognosis of TMC has been confirmed. These studies were, however, rather small with 26 TMC in one study and 24 TMC in the other, and in none of the studies was a control group of 'ordinary' infiltrating duct carcinomas included.

In a recent study of 131 breast carcinomas with medullary features we have, adopting the criteria put forward by Ridolfi *et al.* (1977) and Fisher *et al.* (1975), analysed the inter- and intraobserver variability in the diagnosis of MC, and its prognostic implications (Pedersen *et al.*, 1989). We found a considerable inter- as well as intraobserver variation with significant clinical implications regarding prognosis. The study indicated that the criteria of MC need to be sharpened and simplified in order to increase reproducibility.

The present analysis is part of an extensive study, conducted in collaboration with the Danish Breast Cancer Cooperative Group (DBCG), of histopathological, clinical and biochemical charcteristics of MC. A proposal for a new simplified histopathological definition of MC is presented, based on the study of inter- and intraobserver variability and prognostic importance of 11 histopathological characteristics in a material of breast cancers with medullary features.

Correspondence: L. Pedersen, Department of Oncology ONK, Rigshospitalet 9, Blegdamsvej, DK - 2100 Copenhagen, Denmark.
Received 19 July 1990; and in revised form 26 November 1990.

### Materials and methods

The histopathological material was similar to that used in the study of inter- and intraobserver variability in the diagnosis of MC (Pedersen *et al.*, 1989). Briefly, it consisted of 131 breast carcinomas with medullary features, registered in the DBCG from 1977–1982. Four micron thick sections, obtained from 2–6 paraffin blocks from each tumour, were stained with haematoxylin/eosin and van Gieson. Each set of slides was assessed independently by two senior pathologists, both specialists in breast pathology. The slides were evaluated for the 11 histopathological features described in Table I. The slides were primarily evaluated at leisure over a few months, and in order to establish the intraobserver variability they were re-examined 6–12 months later independently by both pathologists. They were not recoded, but the first diagnoses were concealed from the pathologists. Regarding syncytial growth, the definition was based on the one used by Ridolfi *et al.* (1977), 'broad, interanastomosing sheets of tumour cells.' Tumour growth pattern was characterised as predominantly syncytial when $>75\%$ of the tumour grew in this fashion (Ridolfi *et al.*, 1977). As to mitosis, it was evaluated in ten high power fields (HPF, $d \sim 1$ mm), ($\times 400$). As to mononuclear cells, these were defined as lymphocytes and plasma cells, and were not further subdivided. Regarding histological malignancy grading, the recommendations of WHO (1968) were adhered to. After histopathological evaluation the specimens were assigned to one of three diagnostic alternatives: TMC, AMC or NMC (non-medullary carcinoma) as defined by Ridolfi *et al.* (1977). Figure 1 shows a picture of a typical medullary carcinoma of the breast, illustrating the syncytical growth pattern and the stroma, which is heavily infiltrated with lymphocytes. Results of the diagnostic assessment have been presented recently (Pedersen *et al.*, 1989).

### *Statistical methods*

For each of the histopathological characteristics, overall agreement and Kappa value (Cohen, 1960; Gjørup & Jensen, 1986) were calculated for the two assessments of the individual pathologist (intraobserver agreement) and for the first and second set of assessments by the two pathologists (inter-

**Table I** Histopathological characteristics analysed

| | |
|---|---|
| (1) Growth pattern | Predominantly syncytial (>75%)<br>Non-syncytial |
| (2) Circumscription | Completely circumscribed<br>Monofocal infiltration<br>Multifocal infiltration |
| (3) Stromal component | Sparse<br>Moderate<br>Marked |
| (4) Grade of mononuclear infiltrate | Sparse<br>Moderate<br>Marked |
| (5) Distribution of mononuclear infiltrate | Diffuse<br>Focal<br>Limited to borders only |
| (6) Intraductal component | Present<br>Absent |
| (7) Tubular component | Present<br>Absent |
| (8) Mitoses | <2 per high power field<br>2–3 per high power field<br>>3 per high power field |
| (9) Nuclear pleomorphism | Slight<br>Moderate<br>Marked |
| (10) Histological grade | Grade 1<br>Grade 2<br>Grade 3 |
| (11) Necrosis | Sparse<br>Moderate<br>Marked |

**Figure 1** Typical medullary carcinoma of the breast in low power view. Notice the syncytial growth and the heavy infiltration with lymphocytes.

observer agreement). The prognostic importance of the individual histopathological characteristics was analysed by Kaplan-Meier plots of the subgroups (RFS and overall survival (OS)), which were compared by log rank tests. Cox regression model for survival data was not applied, primarily because of the limited size of the population.

In comparing the diagnoses of the two pathologists, we have used histopathological material from all patients registered from 1977–1982 as having medullary carcinoma of the breast. In the prognostic observations we have limited the inclusion to the patients who were eligible for entry into the adjuvant DBCG protocols. Organisation, design, and follow-up of the DBCG programme and the 77-protocols have been described in detail elsewhere (Andersen *et al.*, 1981).

The histopathological parameters with prognostic significance and an acceptable inter- and intraobserver reproducibility were consequently combined to form a new set of diagnostic criteria for medullary carcinoma of the breast ('New' MC). Using this set of criteria, diagnostic inter- and intraobserver variation has been calculated in relation to each set of the four histopathological evaluations (two evaluations by each pathologist). To evaluate the prognostic importance of this new set of diagnostic criteria, survival curves have been drawn and compared by log rank tests for 'new' MC, the corresponding group of NMC, a control group of infiltrating duct carcinomas grade II + III, and a control group of all infiltrating duct carcinomas registered and protocolled in the DBCG during the same period (1977–1982). The prognostic observations and the data on diagnostic inter- and intraobserver variability of 'new' MC were compared to corresponding data for TMC as defined by Ridolfi.

Finally, a 'final diagnosis' of MC (and of TMC and AMC) was achieved by stressing the diagnoses on which there was agreement in three or four of the four evaluations (two evaluations by each pathologist). Regarding the remaining specimens the pathologists met over a double microscope and came to an agreement about the diagnoses. Kaplan-Meier plots and log rank tests have then been performed on these 'final diagnoses' of MC, a control group of IDC grade II + III, a control group of all IDC, and on the 'final diagnoses' of TMC and AMC.

### Results

Inter- and intraobserver agreement for the 11 histopathological characteristics is given in Table II. Median interobserver overall agreement in the first evaluation was 81% with a range from 34% to 87%, and a median Kappa value of 0.46 (range: 0.05–0.61). Median interobserver overall agreement for the second evaluation was 74% (range: 62–87%), and median Kappa value was 0.36 (range: 0.21–0.68). Regarding intraobserver agreement, median overall agreement for pathologist 1 was 90% (range 68–95%) with a median Kappa value of 0.69 (range: 0.46–0.80). For pathologist 2, median intraobserver overall agreement was 74% (range: 47–91%) with an median Kappa value of 0.44 (range 0.09–0.57).

Regarding prognostic importance of the histopathological features, only a few parameters had a continuous prognostic importance in the four histopathological evaluations. Significantly prognostic $P$-values ($<0.05$) and trends ($0.05 \leqslant P < 0.15$) for the 11 histopathological characteristics are given in Table III. Syncytial growth pattern seems to indicate a better prognosis than a non-syncytial growth pattern. A less favourable outcome for patients with a sparse mononuclear infiltrate than for patients with a marked or a moderate infiltrate was noticed. In Table III the last two components have therefore been combined in one prognostic category. Tumours with diffuse mononuclear infiltration had in all four histopathological evaluations a better prognosis than tumours with focal infiltration, or infiltration limited to borders only, the last two groups have been combined in one group in Table III. As to intraductal component, this feature seems to indicate a poor prognosis. Necrosis also seems to carry some prognostic impact, sparse necrosis indicating the best prognosis. As to stromal component, this might have a prognostic importance. However, these evaluations comprised merely three and two tumours with marked stroma, respectively. Circumscription, tubular component, mitotic rate, nuclear pleomorphism, and histological grade did not seem to have any prognostic significance in this patient material. However, we want to stress that in the few cases with significant $P$-values for histological grade, nuclear pleomorphism, tubular component, tumours with low histological differentiation (high histological grade) pursued a more benign course, contrary to what has been described concerning infiltrating duct carcinomas (Bloom & Richardson, 1957; Rank *et al.*, 1987; Davis *et al.*, 1986; Fisher *et al.*, 1986).

Based on the above described observations, a new simplified histopathological definition of MC is proposed. The criteria include: (1) syncytial growth (>75%) and (2) diffuse,

**Table II** Inter- and intraobserver agreement for 11 histopathological characteristics in 131 breast carcinomas with medullary features

| | *Interobserver agreement* | | | | *Intraobserver agreement* | | | |
|---|---|---|---|---|---|---|---|---|
| | *First evaluation* | | *Second evaluation* | | *Pathologist 1* | | *Pathologist 2* | |
| *Histopathological criteria* | *Overall agreement %* | *Kappa value* | *Overall agreement %* | *Kappa value* | *Overall agreement %* | *Kappa value* | *Overall agreement %* | *Kappa value* |
| Growth | 87 | 0.45 | 85 | 0.59 | 95 | 0.80 | 79 | 0.35 |
| Circumscription | 57 | 0.21 | 69 | 0.43 | 68 | 0.47 | 56 | 0.23 |
| Stroma | 87 | 0.58 | 78 | 0.36 | 90 | 0.46 | 84 | 0.57 |
| Mononuclear infiltrate | | | | | | | | |
| Grade of infiltration | 72 | 0.54 | 71 | 0.51 | 81 | 0.70 | 69 | 0.48 |
| Distribution | 73 | 0.44 | 74 | 0.33 | 85 | 0.65 | 75 | 0.46 |
| Intraductal component | 83 | 0.47 | 84 | 0.53 | 86 | 0.54 | 81 | 0.37 |
| Tubular component | 80 | 0.21 | 86 | 0.21 | 92 | 0.69 | 91 | 0.45 |
| Mitoses | 85 | 0.53 | 67 | 0.23 | 91 | 0.70 | 75 | 0.44 |
| Nuclear pleomorphism | 34 | 0.05 | 62 | 0.24 | 91 | 0.70 | 47 | 0.09 |
| Histological grade | 81 | 0.46 | 72 | 0.29 | 93 | 0.74 | 72 | 0.38 |
| Necrosis | 82 | 0.61 | 87 | 0.68 | 85 | 0.63 | 78 | 0.51 |

**Table III** *P*-values for prognostic observations on 11 histopathological characteristics in 131 breast carcinomas with medullary features. For each histopathological parameter Kaplan-Meier plots of recurrence-free survival (RFS) and overall survival (OS) have been drawn for the subgroups and compared by log rank tests.

| | *First evaluation* | | | | *Second evaluation* | | | |
|---|---|---|---|---|---|---|---|---|
| | *Pathologist 1* | | *Pathologist 2* | | *Pathologist 1* | | *Pathologist 2* | |
| *Histopathological parameter* | *RFS* | *(OS)* | *RFS* | *(OS)* | *RFS* | *(OS)* | *RFS* | *(OS)* |
| Growth | 0.004 | (0.12) | 0.13 | (N.S.) | N.S. | (N.S.) | 0.08 | (0.14) |
| Circumscription | N.S. | (N.S.) | N.S. | (N.S.) | N.S. | (N.S.) | N.S. | (0.13) |
| Stroma | 0.05 | (N.S.) | N.S. | (N.S.) | 0.11 | (0.07) | 0.0001 | (0.009) |
| Grade of mononuclear infiltration | 0.002 | (0.07) | 0.003 | (N.S.) | 0.08 | (N.S.) | 0.05 | (N.S.) |
| Distribution of mononuclear infiltration | 0.04 | (N.S.) | 0.002 | (0.09) | 0.03 | (N.S.) | 0.12 | (N.S.) |
| Intraductal component | N.S. | (N.S.) | 0.004 | (N.S.) | 0.05 | (N.S.) | N.S. | (N.S.) |
| Tubular component | N.S. | (N.S.) | 0.10 | (0.05) | N.S. | (N.S.) | N.S. | (N.S.) |
| Mitoses | N.S. | (0.12) | N.S. | (N.S.) | N.S. | (N.S.) | N.S. | (N.S.) |
| Nuclear pleomorphism | N.S. | (N.S.) | N.S. | (N.S.) | 0.09 | (N.S.) | N.S. | (N.S.) |
| Histological grade | N.S. | (N.S.) | 0.007 | (N.S.) | 0.12 | (N.S.) | N.S. | (N.S.) |
| Necrosis | N.S. | (0.05) | 0.04 | (0.09) | N.S. | (N.S.) | 0.03 | (0.004) |

NS = $P \geqslant 0.15$

moderate or marked mononuclear infiltration. Diagnostic inter- and intraobserver agreement using these criteria are summarised in Table IV, and for comparison the diagnostic inter- and intraobserver variation, using the criteria of TMC and AMC as proposed by Ridolfi, is given in Table V (Pedersen *et al.*, 1989). As can be seen, both the diagnostic inter- and intraobserver agreement have in our hands been considerably improved with this novel approach. Table VI expresses the prognostic importance of the new definition of MC and of Ridolfi's TMC, by comparing survival curves for 'new' MC and for TMC to survival curves for the corresponding NMC, infiltrating duct carcinomas grade II + III, and all infiltrating duct carcinomas registered in the DBCG from 1977–1982. It is obvious that 'new' MC has a better prognosis than the control groups. The prognostic importance of TMC is generally somewhat smaller and more uneven. It is noteworthy that the prognostic importance of MC was not improved by including the criteria of 'no intraductal component' or of 'only sparse to moderate necrosis' in the diagnostic set of histopathological criteria for MC. Furthermore, the inter- and intraobserver agreement deteriorated in both instances when including these parameters. Stroma has not been included, as it had no importance when growth was included.

Survival curves for the final diagnosis of 'new' MC, NMC, and IDC grade II + III appear in Figures 2 and 3. For comparison, the corresponding survival curves for the final diagnosis of TMC and AMC are given in Figures 4 and 5. In all figures, the number of patients at risk is indicated under the abcissa. Notice that the group of 'new' MC, based on the more simple histopathological definition, comprises almost twice the number of patients as the group of TMC. Both RFS and OS are significantly better for MC than for IDC grade II + III. When comparing MC to all IDC, corresponding trends in favour of MC are seen. Corresponding trends are seen for TMC, but no significant *P*-values appear when comparing TMC to either IDC grade II + III or to all IDC.

**Table IV** Inter- and intraobserver agreement of pathologists 1 and 2 in the diagnosis of medullary and non-medulllary carcinoma of the breast, according to new criteria

| | *Overall agreement %* | *Kappa value* |
|---|---|---|
| First evaluation of pathologist 1 *versus* first evaluation of pathologist 2 | 80 | 0.59 |
| Second evaluation of pathologist 1 *versus* second evaluation of pathologist 2 | 84 | 0.67 |
| First evaluation of pathologist 1 *versus* second evaluation of pathologist 1 | 89 | 0.78 |
| First evaluation of pathologist 2 *versus* second evaluation of pathologist 2 | 80 | 0.59 |

**Table V** Inter- and intraobserver agreement of pathologists 1 and 2 in the diagnosis of Typical, Atypical and Non-medullary carcinoma of the breast, using the criteria of Ridolfi *et al.*

| | *Overall agreement %* | *Kappa value* |
|---|---|---|
| First evaluation of pathologist 1 *versus* first evaluation of pathologist 2 | 72 | 0.55 |
| Second evaluation of pathologist 1 *versus* second evaluation of pathologist 2 | 68 | 0.52 |
| First evaluation of pathologist 1 *versus* second evaluation of pathologist 1 | 77 | 0.64 |
| First evaluation of pathologist 2 *versus* second evaluation of pathologist 2 | 63 | 0.44 |

**Table VI** $P$-values for the two histopathological evaluations, comparing with the log rank test the prognosis (Kaplan-Meier plots of recurrence-free survival (RFS) and overall survival (OS)) for medullary carcinoma of the breast based on the new definition ('new' MC) to the prognosis of (1) the corresponding non-medullary carcinoma (NMC), (2) infiltrating duct carcinoma (IDC) grade II + III, and (3) all IDC registered and protocolled in the DBCG from 1977–1982. In the last three items the corresponding values for typical medullary carcinoma (TMC) as defined by Ridolfi

| | *First evaluation* | | | | *Second evaluation* | | | |
|---|---|---|---|---|---|---|---|---|
| | *Pathologist 1* | | *Pathologist 2* | | *Pathologist 1* | | *Pathologist 2* | |
| | *RFS* | *(OS)* | *RFS* | *(OS)* | *RFS* | *(OS)* | *RFS* | *(OS)* |
| 'New' MC *versus* NMC | 0.02 | (0.28) | 0.07 | (0.43) | 0.02 | (0.60) | 0.05 | (0.18) |
| 'New' MC *versus* IDC* gr. II + III | 0.01 | (0.05) | 0.01 | (0.08) | 0.03 | (0.10) | 0.02 | (0.04) |
| 'New' MC *versus* all IDC | 0.07 | (0.22) | 0.14 | (0.31) | 0.07 | (0.36) | 0.09 | (0.15) |
| TMC *versus* NMC | 0.004 | (0.30) | 0.07 | (0.38) | 0.19 | (0.78) | 0.27 | (0.62) |
| TMC *versus* IDC gr. II + III | 0.02 | (0.05) | 0.04 | (0.02) | 0.04 | (0.23) | 0.28 | (0.32) |
| TMC *versus* all IDC | 0.07 | (0.16) | 0.15 | (0.07) | 0.13 | (0.57) | 0.60 | (0.70) |

*IDC: infiltrating duct carcinoma of the breast.

**Figure 2** Kaplan-Meier plots concerning recurrence-free survival (RFS) of the final diagnoses of medullary carcinoma of the breast based on the new histopathological definition ('new' MC), non-medullary carcinoma of the breast (NMC), and infiltrationg duct carcinoma grade II + III (IDC gr. II + III). The number of patients at risk is indicated under the abcissa.

**Figure 4** Kaplan-Meier plots concerning RFS of the final diagnosis of typical medullary carcinoma of the breast (TMC) (Ridolfi's definition), atypical medullary carcinoma of the breast (AMC), NMC, and infiltrating duct carcinoma grade II + III (IDC gr. II + III). The number of patients at risk is indicated under the abcissa.

**Figure 3** Kaplan-Meier plots concerning overall survival (OS) of the final diagnoses of 'new' MC, NMC and infiltrating duct carcinoma grade II + III (IDC gr. II + III). The number of patients at risk is indicated under the abcissa.

**Figure 5** Kaplan-Meier plots concerning OS of the final diagnoses of TMC, AMC, NMC, and infiltrating duct carcinoma (IDC gr. II + III). The number of patients at risk is indicated under the abscissa.

### Discussion and conclusion

If a histopathological diagnosis of breast cancer is to be of value to the physician, certain criteria must be fulfilled. The diagnostic criteria must be uniform, the individual observers must be consistent in their diagnoses, and different observers must agree in their diagnoses. It is further desirable to render a histologic diagnosis with prognostic influence. These demands also apply to the single histopathological features on which the diagnosis is based. In histopathology, quality control is necessary, as the subjectivity in judging the specimens can be substantial (Langley, 1978). This study was designed to elucidate the reproducibility of 11 histopathological characteristics in 131 breast cancers with medullary features, and to evaluate the prognostic importance of the histopathological parameters and the influence of inter- and intraobserver variability on prognosis. The best interobserver reproducibility was observed for growth pattern (syncytial/non-syncytial) and grade of necrosis. The lowest interobserver reproducibility was registered for nuclear pleomorphism. In the literature, only a few studies have dealt with reproducibility of histopathological characteristics in breast cancer. The parameters most often assessed are histological or nuclear grading, including tubule formation, mitoses, nuclear pleomorphism, and hyperchromatic nuclei (Delides *et al.*, 1982; Stenkvist *et al.*, 1979; Culter *et al.*, 1966; Gilchrist *et al.*, 1985; Fisher, 1985; Bloom & Richardson, 1957). On the whole, our data on interobserver agreement on histological grade and appertaining histopathological parameters are equal to or better than those quoted in the literature, but still disappointingly low. Interobserver variability concerning grade of lymphocytic infiltration in breast cancer was investigated in a UICC study (1978). Results from that study and the present study are very much alike.

Also concerning intraobserver agreement, our data on histological grade and appertaining histopathological parameters are equal to or better than those quoted in the literature (Stenkvist *et al.*, 1979; Cutler *et al.*, 1966; Fisher, 1985). Again, most of the histopathological features have not been evaluated previously in the literature as to reproducibility.

If a prognostic histopathological factor is to be of value in the therapeutic management of patients with breast cancer, the prognostic implications of inter- and intraobserver variability must be without significance. In other words, the prognostic importance of a given histopathological factor should be consistent. In our study, grade of mononuclear infiltration and distribution of mononuclear infiltrate had continuous prognostic importance in the four histopathological evaluations. Also, growth pattern, stromal component, and necrosis seemed to have prognostic importance. On the other hand, circumscription, which in previous articles has been emphasised as one of the morphological markers of MC (Pedersen *et al.*, 1988; Fisher *et al.*, 1975; Ridolfi *et al.*, 1977; Foote & Stewart, 1946; Haagensen, 1973; Richardson, 1956; McDivitt *et al.*, 1968; Schwartz, 1969), had not prognostic significance in our study. Nor did features concerning histological grade have any prognostic importance. Ridolfi *et al.* (1977) and Wargotz and Silverberg (1988) also found the intensity of mononuclear stromal infiltration to be correlated with prognosis in each of the three diagnostic subgroups (TMC, AMC, and NMC). They, too, found that microscopic distinctions between minimal and multifocal infiltrative margins did not appreciably alter prognosis and that intraductal carcinoma did not appreciably affect survival. The material of Wargotz *et al.* was very small, however.

Based on the previous data we propose a simplified histopathological definition of MC: (1) syncytial growth pattern and (2) diffuse, moderate or marked mononuclear infiltration. With these criteria, diagnostic reproducibility of MC is acceptable, and prognostic importance is significant and not influenced by diagnostic inter- and intraobserver variability. Furthermore, it should be stressed that both inter- and intraobserver agreement, as well as prognostic importance have been improved compared to the results obtained with Ridolfi's definition of TMC and AMC applied on the same tumour and patient material (1977). Before it is generally accepted, this simplified histopathological definition of MC should be tested as regards prognosis in another population of patients with breast cancer.

Supported by The Danish Cancer Society. We further wish to thank the Departments of Pathology in Denmark for the supply of histological material.

### References

ANDERSEN, K.W., MOURIDSEN, H.T., CASTBERG, T. & 8 others (1981). Organisation of the Danish adjuvant trials in breast cancer. *Dan. Med. Bull.*, **28**, 102.

BLOOM, H.J.G. & RICHARDSON, W.W. (1957). Histological grading and prognosis in breast cancer. A study of 1409 cases of which 359 have been followed for 15 years. *Br. J. Cancer*, **11**, 359.

COHEN, J. (1960). A coefficient of agreement for nominal scales. *Educat. Psychol. Measurement*, **20**, 37.

CUTLER, S.J., BLACK, M.M., FRIEDELL, G.H., VIDONE, R.A. & GOLDENBERG, I.S. (1966). Prognostic factors in cancer of the female breast. II. Reproducibility of histopathologic classification. *Cancer*, **19**, 75.

DAVIS, B.W., GELBER, R.D., GOLDHIRSCH, A. & 8 others (1986). Prognostic significance of tumor grade in clinical trials of adjuvant therapy for breast cancer with axillary lymph node metastasis. *Cancer*, **58**, 2662.

DELIDES, G.S., GARAS, G., GEORGOULI, G. & 4 others (1982). Intralaboratory variations in the grading of breast carcinoma. *Arch. Pathol. Lab. Med.*, **106**, 126.

FISHER, B., FISHER, E.R., REDMOND, C., BROWN, A. & CONTRIBUTING NSABP INVESTIGATORS (1986). Tumor nuclear grade, estrogen receptor, and progesterone receptor: their value alone or in combination as indicators of outcome following adjuvant therapy for breast cancer. *Breast Cancer Res. Treat.*, **7**, 147.

FISHER, E., GREGORIO, R.M. & FISHER, B. (1975). The pathology of invasive breast cancer (National Surgical Adjuvant Breast Project). *Cancer*, **36**, 1.

FISHER, E.R. (1985). Comment on 'Interobserver reproducibility of histopathological features in stage II breast cancer' by K.W. Gilchrist *et al. Breast Cancer Res. Treat.*, **5**, 11.

FOOTE, F.W. & STEWART, F.W. (1946). A histological classification of carcinoma of the breast. *Surgery*, **19**, 74.

GILCHRIST, K.W., KALISH, L., GOULD, V.E. & 10 others (1985). Interobserver reproducibility of histopathological features in stage II breast cancer. *Breast Cancer Res. Treat.*, **5**, 3.

GJØRUP, T. & JENSEN, A.M. (1986). Kappakoefficienten – et mål for reproducerbarhed af nominale og ordinale data. *Nord. Med.*, **101**, 90.

HAAGENSEN, C.D. (1973). *Diseases of the Breast*. W.B. Saunders (2nd ed): Philadelphia.

LANGLEY, F.A. (1978). Quality control in histopathology and diagnostic cytology. *Histopathology*, **2**, 3.

LANDIS, J.R. & KOCH, G.G. (1977). The measurement of observer agreement for categorical data. *Biometrics*, **33**, 159.

MCDIVITT, R.W., STEWART, F.W. & BERG, J. (1968). *Tumors of the Breast: Atlas of Tumor Pathology*. Armed Forces Institute of Pathology, Second Series, Fascicle 2, pp. 57–66: Washington D.C.

PEDERSEN, L., HOLCK, S. & SCHIØDT, T. (1988). Medullary carcinoma of the breast. *Cancer Treat. Rev.*, **15**, 53.

PEDERSEN, L., HOLCK, S., SCHIØDT, T., ZEDELER, K. & MOURIDSEN, H.T. (1989). Inter- and intraoberver variability in the histopathological diagnosis of medullary carcinoma of the breast, and its prognostic implications. *Breast Cancer Res. Treat.*, **14**, 91.

RANK, F., JESPERSEN, N.C.B., PEDERSEN, B.V., KEIDING, N. & DOMBERNOWSKY, P. (1987). Histological malignancy grading of invasive ductal breast carcinoma. *Cancer*, **60**, 1299.

RAPIN, V., CONTESSO, G., MOURIESSE, H. & 6 others (1988). Medullary breast carcinoma. A reevaluation of 95 cases of breast cancer with inflammatory stroma. *Cancer*, **61**, 2503.

RICHARDSON, W.W. (1956). Medullary carcinoma of the breast. *Br. J. Cancer*, **10**, 415.

RIDOLFI, R.L., ROSEN, P.P., PORT, A., KINNE, D. & MIKE, V. (1977). Medullary carcinoma of the breast. *Cancer*, **40**, 1365.

SCHWARTZ, G.F. (1969). Solid circumscribed carcinoma of the breast. *Ann. Surg.*, **169**, 165.

STENKVIST, B., WESTMAN-NAESER, S., VEGELIUS, J. & 4 others (1979). Analysis of reproducibility of subjective grading systems for breast carcinoma. *J. Clin. Pathol.*, **32**, 979.

UICC (1978). *An International Survey of Distributions of Histologic Types of Breast Cancer*. Correa, P. & Johnson, W.D. (eds), pp. 24, 211–215. International Union Against Cancer: Geneva.

WARGOTZ, E.S. & SILVERBERG, S.G. (1988). Medullary carcinoma of the breast – a clinicopathologic study with appraisal of current diagnostic criteria. *Hum. Pathol.*, **19**, 1340.

WHO (1968). *Histological Typing of Breast Tumours*. Scarff, R.W. & Torloni, H. (eds). Worth Health Organization: Geneva.

WHO (1982). The World Health Organization Histological Typing of Breast Tumours – second edition. *Am. J. Clin. Pathol.*, **78**, 806.